# Nitrate of Silver in the Treatment of Abscesses

**Published:** 1849-08

**Authors:** 


*Nitrate of Silver in the Treatment of Abscesses.* By M. Nonat.—In abscesses, where it is desirable to prevent union of the parts, gentle cauterization of the edges of the wound with nitrate of silver in substance, repeated at intervals of one, two, or three days, is, in the opinion of M. Nonat, of the Cochin hospital, preferable to the use of pledgets of lint. It is more cleanly, less painful, not so likely to be followed by unpleasant consequences, and much easier of application than the lint.—*Ibid.*

